# Pneumoperitoneum in a COVID-19 Patient Due to the Macklin Effect

**DOI:** 10.7759/cureus.13200

**Published:** 2021-02-07

**Authors:** Ramon Vidrio Duarte, Eduardo Vidrio Duarte, Juan Gutierrez Ochoa, Maria Camila Gaviria Leiva, Joaquin A Pimentel-Hayashi

**Affiliations:** 1 General Surgery, Hospital General de México "Dr. Eduardo Liceaga", Mexico City, MEX; 2 General Surgery, Hospital Angeles Metropolitano, Mexico City, MEX; 3 Department of Anaesthesiology, Hospital General de México "Dr. Eduardo Liceaga", Mexico City, MEX; 4 Allergy and Immunology, World Allergy Organization (WAO) Center of Excellence, Hospital Infantil de México Federico Gómez, Mexico City, MEX

**Keywords:** barotrauma, covid-19, macklin effect, pneumoperitoneum

## Abstract

A 63-year-old male with coronavirus disease 2019 (COVID-19) pneumonia presented to the emergency department, supplementary oxygen is delivered via nasal cannula, and invasive ventilation was not needed; there was significant pneumoperitoneum on radiologic control. After a meticulous examination of the thoracic tomography, there were some linear air collections adjacent to the bronchovascular sheaths, indicative of the Macklin effect, without abdominal alterations, and the patient remained stable; therefore, we did not perform a surgical procedure, and the pneumoperitoneum reabsorbed spontaneously on radiologic control.

The pulmonary origin of pneumoperitoneum is unusual and is associated with mechanical ventilation and alveolar leak; the air leak with subsequent dissection into other anatomical spaces is called the Macklin effect. It is essential to have this mechanism in mind because most of these patients respond well to conservative treatment. When studying primary pneumoperitoneum, the cause should be studied carefully to discard visceral perforation, tracheal or esophageal rupture.

## Introduction

Pneumoperitoneum is an abnormal collection of air in the peritoneal cavity; the most common cause is postsurgical pneumoperitoneum; excluding this cause, approximately 85% correspond to visceral perforation, mostly by a gastric or duodenal ulcer. Pseudo-pneumoperitoneum includes adventitial air shadows, over distention of hollow viscera, gas trapped within wounds, and Chilaiditi’s sign [1].

Pneumoperitoneum without visceral perforation, or primary, accounts for 5% to 15%, most of these cases do not require surgical management; in these cases, the underlying cause has to be determined. Amongst the most common abdominal etiologies are peritoneal dialysis, endoscopic gastrointestinal procedures, gastrostomy status, spontaneous bacterial peritonitis, and least common, pneumatosis cystoides intestinalis. There are also thoracic causes of pneumoperitoneum, such as trauma, cardiopulmonary resuscitation, tracheal rupture, or pneumothorax. Several authors have investigated the relationship between mechanical ventilation and pneumoperitoneum development, mainly when high pressures are employed [1,2].

We present a case of pneumoperitoneum secondary to supplementary oxygen delivery via nasal cannula in the context of the coronavirus disease 2019 (COVID-19) pandemic and its surgical implications as a word of caution to other possible cases worldwide.

## Case presentation

A 63-year-old male with obesity and a four-year history of type II diabetes, along with a 10-year history of hypertension, was admitted to the emergency department; he started eight days before his admission with nausea, headache, fever, malaise, and dry cough. A day before his admission, he developed dyspnea. His vital signs were temperature 38^o^C, oxygen saturation (SO2) 80% under ambient air, blood pressure (BP) 100/60 mmHg, heart rate 90 bpm, respiratory rate of 28 breath/min, oxygen saturation raised to 85% with nasal cannula supplementation at 4 L/min and later to 93% at 6 L/min; therefore he was admitted to the COVID-19 unit at our institution.

**Figure 1:**
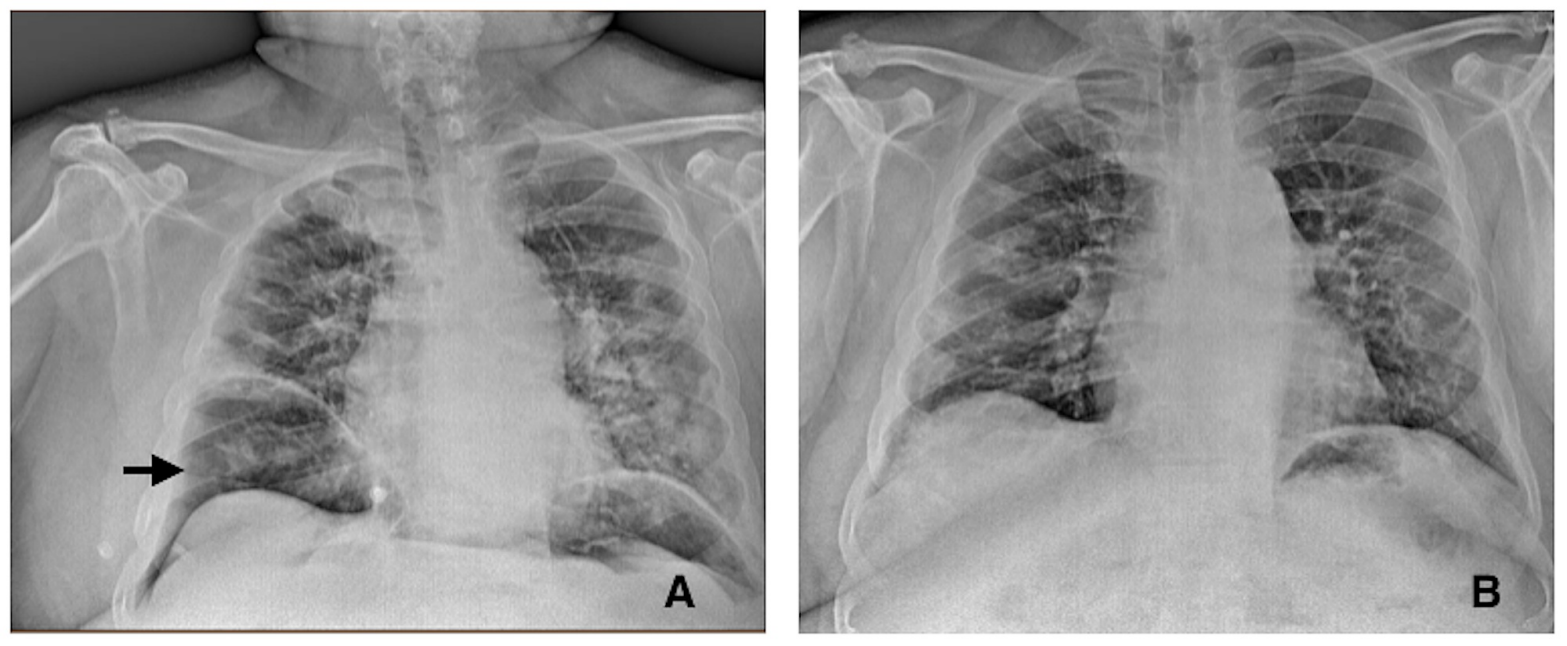
Chest radiograph. A: Index radiography with diffuse bilateral opacities, with subdiaphragmatic free air (arrow); B: Discharge radiography  with pneumoperitoneum resorption and diminish of the pulmonary inflammatory process.

**Figure 2:**
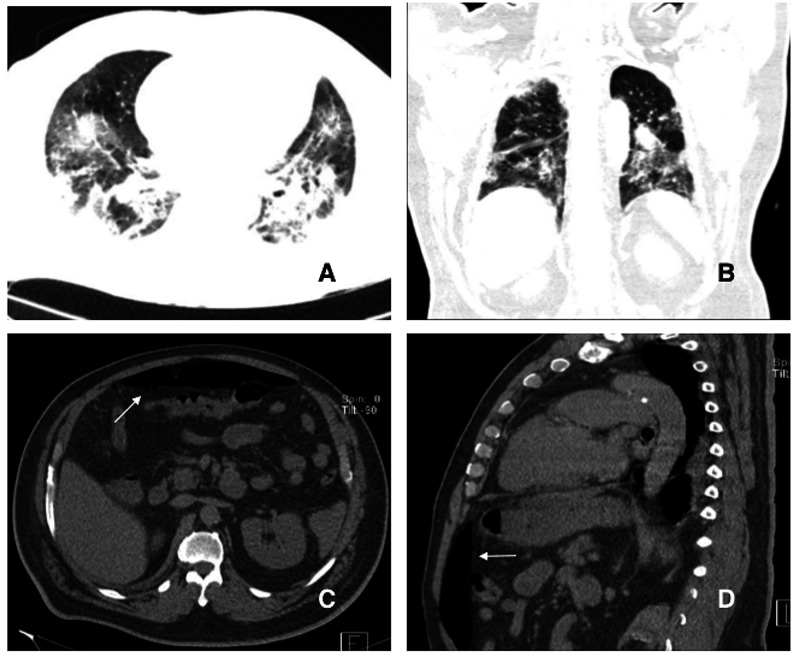
CT images of the chest and abdomen. Panels A and B: Diffuse ground-glass opacities in both lungs; Panels C and D: Abdomen images showing pneumoperitoneum (arrows).

**Figure 3:**
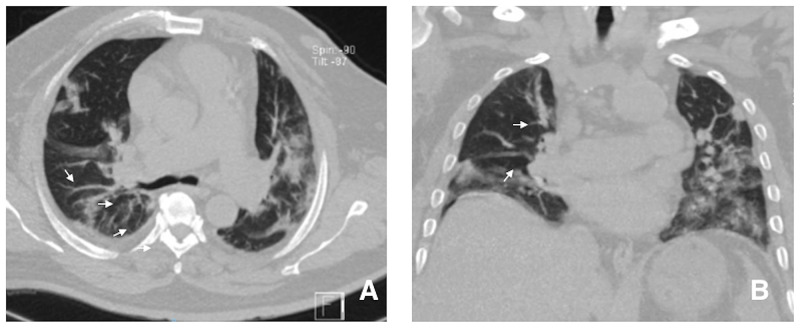
CT images of the chest compatible with the Macklin effect. Ground-glass images and linear air collections indicative of air leak (arrows).

Laboratory tests showed an elevated C-reactive protein at 90.4 mg/L, ferritin 388 ng/ml, D-Dimer 9484 ug/L, leucocytes 7.8 x10^3^/uL, with no other test abnormality. Initial X-Ray showed bilateral, multifocal, and peripheral infiltration with a ground-glass pattern, along with apparent subdiaphragmatic free air (Figure 1); the patient remained stable using a face mask with a reservoir. The thoracic tomography showed multiple patchy areas of ground-glass opacity, interlobular septal thickening; these lesions were mainly localized peripheral and in the posterior part of the lungs, along with pneumoperitoneum. Therefore, we performed a complete abdominal contrasted tomography; with no evidence of visceral perforation or other possible cause of the gas collection in the peritoneal cavity (Figure 2). After a meticulous examination of the thoracic tomography, some linear air collections were visualized adjacent to the bronchovascular sheaths as a radiological sign of the Macklin effect; no mediastinal or retroperitoneal air collections were identified (Figure 3).

No pathological findings were found in a thorough abdominal examination. Due to the lack of abdominal signs or radiologic findings of visceral perforation, we did not perform any surgical treatment; the surgical team closely observed the patient, who continued eating with no defecatory alterations, and the abdominal pressure remained within the normal range. After three days, our institution's epidemiology department confirmed that the nasopharyngeal swab test was positive for severe acute respiratory syndrome coronavirus 2 (SARS-CoV-2) by real-time reverse transcription polymerase chain reaction (rRT-PCR) assay.

On the seventh day, the X-ray showed remission of the inflammatory process and pneumoperitoneum resorption. The patient had no fever for 72 hours, and his respiratory symptoms improved, with no further laboratory findings, SaO2 of 90% under ambient air; therefore, we decided on patient discharge.

## Discussion

The thoracic origin of pneumoperitoneum is mostly associated with mechanical ventilation; nonetheless, with the novel COVID-19 pandemic, this radiological finding might present in some patients, especially with high-pressure management, along with the fact that many doctors with little expertise in the management of mechanical ventilation devices are nowadays treating these patients. Therefore, in the context of a patient with no abdominal manifestations and no inflammatory response, the non-surgical causes of pneumoperitoneum should be considered [3,4].

The Macklin effect is mainly associated with pneumomediastinum, mostly on trauma patients, and on respiratory infections, asthmatic crisis, as a result of prolonged labor, associated with mechanical ventilation or even after a Valsalva maneuver. Also, some case reports associate scuba diving or even basketball or football practice with this phenomenon [5].

The leading cause is alveolar rupture secondary to high pressure leading to air leak; the air centripetally dissects through the pulmonary interstitium along the bronchovascular sheaths causing interstitial emphysema, it might continue dissecting through the interlobular septa in direction to the pulmonary hilum and from there to the mediastinum and sometimes to other anatomical regions [6].

Two different pathways for the passage of air from thoracic to abdominal cavities exist in the literature. The first one is the classically described passage via the mediastinum through the perivascular connective tissue or major diaphragmatic portals to retroperitoneum and finally to the peritoneum; the second pathway is through pleural and diaphragmatic defects, this previous description applies to our case where there is no evidence of pneumomediastinum [7].

It is essential to emphasize the importance of positive end-expiratory pressure (PEEP); patients receiving elevated PEEP have a higher risk of pneumoperitoneum, even above 6 cmH2O. One of the consequences of high PEEP is the "air leak" phenomenon due to an alveolar air passage into the perivascular and peribronchial interstitial tissue, mostly associated with the lung's general condition [8].

The oxygen delivery method employed initially on this patient was a nasal cannula, which is still an optimal method to deliver oxygen on hypoxemic patients. In some emergency rooms during the COVID-19 pandemic, it is the only non-invasive resource to ensure an adequate oxygen saturation level. However, to achieve adequate saturation, a high flow is employed, in spite that a very high flow may lead to barotrauma since it could reach PEEP levels as high as 7.4 cmH20.

The Macklin effect usually appears on computed tomography as linear air collections contiguous to the bronchovascular sheaths, and consequently, air might be present in the mediastinum; multidetector-row CT has reported the highest diagnostic accuracy amongst diagnostic studies [9].

In a SARS-CoV-2 patient context, either positive or suspicious case, care should be taken on deciding whether to perform a surgical procedure due to the high mortality rates. On these patients, and even on asymptomatic patients who underwent elective surgery during the incubation period, mortality is as high as 20.5% [10-12].

A recent article analyzed surgical COVID-19-positive patients; surgical intervention was done in 60.8% of the COVID-19-positive patients, while the rest, 39.2%, were conservatively managed; they reported a 7.6% death rate during the hospital stay [13].

Thus, in the context of a stable patient with no evident surgical cause of pneumoperitoneum, we decided that the safer choice was an expectant treatment with close follow up, quantifying abdominal pressure, since pneumoperitoneum might lead to abdominal compartment syndrome.

## Conclusions

Individual response to SARS-CoV-2 infection is not entirely understood yet, and there is still not enough information on the reaction of these patients to surgical intervention. Thus, the surgeon must rule out SARS-CoV-2 infection to perform an elective surgery safely, but emergency procedures should also be selected carefully. This case is an excellent example of a non-surgical situation that could have taken a different course, and during the COVID-19 pandemic, some other similar cases might appear; we intend to alert surgeons and clinicians of this potential finding due to oxygen supplementation.
